# Clozapine prescribing practice and trends in Qatar: First national observational study

**DOI:** 10.1002/brb3.2617

**Published:** 2022-06-01

**Authors:** Ovais Wadoo, Javed Latoo, Majid Alabdulla, Yassin Eltorki, Sadaf Riaz, Mustafa Abdul Karim, Mohammed Abu‐Hafizah, Shuja Reagu

**Affiliations:** ^1^ Mental Health Services Hamad Medical Corporation Doha Qatar; ^2^ College of Medicine Qatar University Doha Qatar; ^3^ Department of Psychiatry College of Medicine Weill Cornell Medicine Doha Qatar

**Keywords:** antipsychotic prescribing, clozapine, mental health services

## Abstract

**Background:**

Clozapine is the gold standard in the management of treatment‐resistant schizophrenia. Despite its clinically proven efficacy clozapine utilization is variable globally and published evidence is suggestive of its underutilization. Research from the Arab region on clozapine utilization is limited. The aim of our descriptive observational study was to evaluate the prescribing practice of clozapine and its sociodemographic and clinical corelates in the State of Qatar.

**Methods:**

The study is a retrospective case‐note review of all patients maintained on clozapine, in the calendar year 2020. Data were collected on sociodemographic characteristics of the patients; antipsychotic trials before initiating clozapine; and clinical characteristics of the patients, including their diagnoses leading to prescription of clozapine, duration of illness, psychiatric hospitalizations, and co‐morbidities.

**Results:**

During the study period, 100 patients were maintained on clozapine. Patients were mostly Qatari and non‐Qatari Arab males. Prescription rates were significantly different for Qatari patients when compared to non‐Qatari patients. Most patients had a chronic illness with the age of onset of illness in early adulthood and were diagnosed with schizophrenia or schizoaffective disorder. The mean daily dose of clozapine was 325 mg. Eighty percent of the patients received two or more antipsychotic trials before initiating clozapine. Sixty‐eight percent of the patients had more than two antipsychotic trials before initiating Clozapine. One third of patients had no history of psychiatric hospitalizations, and one quarter had five or more previous psychiatric hospitalizations. Of the psychiatric comorbidities, mood and substance use disorders were common. Of medical comorbidities, endocrine and metabolic disorders were common.

**Conclusion:**

Despite apparent underutilization, the Clozapine prescribing rates in Qatar are comparable to countries with plasma monitoring systems when framed within Qatar's unique demographic context. However, there still is a significant delay in Clozapine initiation despite its clinical superiority.

**Strengths and Limitations of this Study:**

- First study on Clozapine utilization from the Middle‐East and North‐Africa region.
- This study examined prescribing of clozapine in a national cohort of patients in Qatar.
- Provides insight into sociodemographic and clinical correlates of clozapine prescribing in a country with 90% migrants.
- Limited by the completeness of the information contained in the patients’ medical charts.

## 1 INTRODUCTION

Schizophrenia is a chronic mental disorder affecting 20 million people worldwide (GBD 2017 Disease and Injury Incidence and Prevalence Collaborators, 2018). Around 30% of these patients suffer from treatment‐resistant schizophrenia (TRS), defined as a lack of response to at least two antipsychotic medications of adequate dose and duration (Lally et al., 2016). Clozapine remains the most effective pharmacological treatment for TRS with 60%–70% response rate (Meltzer, 1992). It is known to improve patient outcomes and prevent hospital admissions. However, clozapine utilization varies by country and has been consistently underutilized despite published clinical evidence supporting clozapine as the gold standard in the treatment of TRS (Alessi‐Severini et al., 2013; Malalagama et al., 2011; Moore et al., 2007; Xu et al., 2020; Wheeler, 2008). Bachmann et al. (2017) report wide variation in clozapine use with utilization being highest in Finland at 0.18% (189.2/100 000 persons) and lowest in Japan at 0.006% (0.6/100 000), with a male preponderance (median male/female clozapine use ratio being 1.5). Clozapine utilization of roughly 0.2% of the population is considered as optimal utilization of clozapine based on prevalence of TRS. The relevant determinants for underutilization and variation in countries may be accounted by patient factors, healthcare professional factors, healthcare system factors, economic factors, cultural factors, and historical factors (Bachmann et al., 2017). These include the diversity of the underlying populations in terms of age and gender, cultural attitudes toward drug treatments, and historical experiences of clozapine in different countries. Clozapine prescribing may also be influenced by licensing regulations, physician's experience in clozapine prescribing, physician's adherence to treatment guidelines, availability and pricing of clozapine, availability and pricing of other antipsychotics, and availability of hematological monitoring (Nielsen et al., 2012; Warnez & Alessi‐Severini, 2014).

At the time of writing this, published research on clozapine utilization and its sociodemographic and clinical corelates from the Arab region is limited. The data about clozapine utilization are mainly derived from literature on antipsychotic prescribing practices. Antipsychotics in the region are commonly used in combination with other psychotropic medications, rather than as monotherapy. Most of the patients are prescribed oral second‐generation antipsychotics (SGAs) in the acute inpatient management of schizophrenia, with Risperidone being most administered in Egypt (44.0%), while Olanzapine being more commonly used in Saudi Arabia (41.4%) and the Gulf States (42.2%). Clozapine was prescribed to 19.1% patients (Alkhadhari et al., 2015; Zaraa et al., 2015). The studies undertaken in the Kingdom of Saudi Arabia (KSA), Oman, and Bahrain reported low utilization of clozapine in patients diagnosed with TRS. In KSA, clozapine was prescribed to 3.1% of all inpatients and 2.4% of all outpatients. In Bahrain and Oman, clozapine was the least prescribed atypical antipsychotic (Al‐Khaja et al., 2012; Alosaimi et al., 2016; Za'abi & Al‐Hinai, 2014). In Qatar, 56.2% of patients prescribed antipsychotics are maintained on SGAs of which 2.4% of patients were maintained on clozapine (Ouanes et al., 2020). Use of first‐generation antipsychotics (FGAs) is limited to 15.5% patients in Qatar, whereas 28.3% were on a combination of FGAs and SGAs. Underutilization of clozapine in the Arab region is predominantly influenced by prescriber‐related barriers (Ismail et al., 2019). Prescribers’ perceptions regarding clozapine's adverse effects and familiarity with the management of those adverse effects and the strict and regular need for hematological monitoring are the predominant identified barriers (Ismail et al., 2019).

Health system barriers have also been identified, including the lack of a nation‐wide monitoring program (Ismail et al., 2019). In many countries in the region, there are no central coordinating agencies to monitor patients on clozapine and hospitals and clinicians make local arrangements. The aim of our descriptive observational study was to describe clozapine prescribing in patients under the care of the public mental health service in Qatar.

## 2 METHODOLOGY

### 2.1 Setting

The State of Qatar is a peninsula amid the Arabian Gulf. It has a population of 2.68 million (Alessi‐Severini et al., 2013). Hamad Medical Corporation (HMC) is the main provider of government‐funded health services, including mental health services (MHS) and caters to the majority of the mental health care provided in the country (Goodman, 2015). A range of services including community, outpatient, and inpatient services are provided free or at subsidized cost. Patients on clozapine are registered under the Medication Therapy Management (MTM) clinic, managed by clinical pharmacists. MTM clinics are coordinating agencies to monitor patients on clozapine. These clinics facilitate blood monitoring and regulate clozapine dispensation. HMC is the only institution in Qatar that has the license to prescribe and dispense clozapine, so any individual on clozapine prescription in Qatar would be captured within this methodology.

### 2.2 Study design, eligible population, and sample

The study design was a retrospective case‐note review. Registers kept by the MTM clinic were reviewed to identify the patients maintained on clozapine. All patients registered with MTM clinic in the calendar year 2020 were included in the study. During the study period, 100 patients were registered out of which 10 were being managed in the inpatient settings and 90 in the community setting. All age groups, nationalities, and both genders were included.

### 2.3 Data extraction and analysis

A standardized data abstraction tool was developed to record relevant information from the electronic patient records (EPR). The initial draft of the data extraction form was first piloted on 30 EPR by three researchers (YT, SR, and MH) to assess feasibility of the tool. Modifications were made to the data collection tool to account for missing or unclear information and a final version was approved after discussion with the wider team.

Data were extracted by three members of the research team (YT, SR, and MH) who held regular discussions to ensure consistency in data recording. Any rating uncertainties were resolved by discussion with a fourth team member (OW). Data collected included sociodemographic characteristics, clinical characteristics, and antipsychotic treatment characteristics. Sociodemographic data included patients’ gender, age, and ethnicity. Data collected on clinical characteristics of the patients included their diagnoses leading to prescription of clozapine, duration of illness, number of psychiatric hospitalizations, and mental and physical comorbidities. Data on medication included the use of other antipsychotics both currently and historically, that is, number and type (SGAs, FGAs) of trials prior to starting clozapine. Details of other co‐prescribed medications were not collected. Mean clozapine dose was derived from total daily doses.

### 2.4 Ethical approval

The study received approval from the HMC Institutional Review Board (IRB) (MRC‐01‐20‐931). Individual patient consent was not deemed necessary by the IRB.

### 2.5 Public and patient involvement

There was no public or patient involvement in the design, conduct, reporting, or dissemination plans of our research.

## 3 RESULTS

A total of 100 patients were identified and reviewed. Ten of them were in the inpatient units and 90 were in the outpatient and community mental health services.

### 3.1 Sociodemographic characteristics

Table 1 shows the sociodemographic details of our sample. Patients were mainly Qatari nationals followed by non‐Qatari Arabs. The mean age was 37 years and the majority (*N* = 68; 68%) were males.

**TABLE 1:** Patient demographic characteristics

Variable			Number of patients
Patient demographic characteristics	Gender	Male	68 (68%)
		Female	32 (32%)
	Age	0–20	3 (3%)
		21–30	30 (30%)
		31–40	36 (36%)
		41–50	23 (23%)
		51–64	4 (4%)
		≥65	4 (4%)
	Nationality	Qatari	53 (53%)
		Non‐Qatari Arabs	26 (26%)
		Indian	7 (7%)
		Pakistani	4 (4%)
		North Americans	4 (4%)
		Other	6 (6%)

### 3.2 Clinical characteristics

Table 2 shows the clinical characteristics of our sample. The overwhelming majority (94%) of patients had a diagnosis of schizophrenia or schizoaffective disorder. The remaining 6% of patients were being treated primarily for bipolar disorder, delusional disorder, and psychotic disorders. Nearly two thirds (*N* = 61; 61%) had no comorbid psychiatric diagnosis. Mood disorders and substance use disorders were common comorbidities in the remaining one‐third patients. Only one patient had a diagnosis of personality disorder and three had intellectual disability. Most patients had a chronic illness with the age of onset of illness in early adulthood. One third (*N* = 33; 33%) of patients had no history of psychiatric admissions, 44 patients (44%) had less than five admissions, and around one quarter (*N* = 23; 23%) of patients had five or more previous psychiatric hospitalizations. Approximately two thirds (*N* = 64; 64%) of the cohort had no physical health diagnosis documented. Of the medical comorbidities, endocrine, cardiovascular, and neurological disorders were present in 21, 13, and seven patients, respectively.

**TABLE 2:** Patient clinical characteristics

Criteria (*n* = 100)			Number of patients
Patient clinical characteristics	Duration of illness (years)	<2	2
		2–5	7
		>5	81
		Unknown	10
	Age at first presentation (years)	0–20	34
		21–30	41
		31–40	5
		41–50	3
		51–64	0
		≥65	0
		Unknown	17
	Main indication for prescription	Treatment‐resistant schizophrenia	81
		Psychosis during the course of Parkinson's disease	0
		Treatment‐resistant schizoaffective	13
		History of neuroleptic malignant syndrome on other antipsychotics	0
		Tardive extrapyramidal side‐effects on other antipsychotics	0
		High‐risk of suicide	0
		Persistent aggressive behaviors	0
		Other	6
	Mental comorbidity	None	61
		Anxiety disorders	7
		Mood disorders	24
		Substances disorders	8
		Personality disorders	1
		LD disorders	3
		Other	0
	Physical comorbidity	None	64
		Cardiovascular	13
		Neurologic	7
		Endocrine	21
		Respiratory	2
		GIT	2
		Other	2
	Number of psychiatric admissions	0	33
		<5	44
		≥5	23

Abbreviations: LD, Learning Disability; GIT, Gastro‐Intestinal Tract.

### 3.3 Clozapine prescribing

Table 3 shows the antipsychotic treatment characteristics of our sample. The mean daily dose of clozapine was 325 mg (range: 25–650 mg/day). Of the 100 patients maintained on clozapine, 58 had no other concomitant antipsychotics prescribed. Forty‐two patients had other concomitant antipsychotics prescribed in addition to clozapine—35% (*N* = 35) were on one concomitant antipsychotic and 7% (*N* = 7) were on two or more concomitant antipsychotics. Of the concomitant antipsychotics, Amisulpride and oral Aripiprazole (*N* = 13 and *N* = 12) were the most used for augmentation. In 13 patients, the augmenting agent was an injectable antipsychotic. In concordance with different guidelines, 12 patients (12%) of our cohort received two antipsychotic trials before initiating clozapine, while three patients (3%) received only one trial and 68 patients (68%) received more than two trials with another antipsychotic before initiating clozapine. In 17% of our patients, we were unable to establish the number of trials prior to clozapine initiation. Table 3 lists the antipsychotic groups trialed before clozapine.

**TABLE 3:** Antipsychotic treatment characteristics

Criteria			Number of patients
Antipsychotic treatment characteristics	Clozapine	Median daily dose (325 mg)	100
	Antipsychotics groups trailed prior to clozapine	None	9
		Oral first generation	57
		Oral second generation	81
		Depot LAI	68
		All	48
		Unknown	8
	Number of antipsychotics trailed prior to clozapine	0	9
		1	3
		2	12
		3	25
		4	16
		≥5	27
		Unknown	8
	Number of concomitant antipsychotics	0	58
		1	35
		2	5
		3	2
		4	0
		≥5	0
	Concomitant Antipsychotics	Amisulpride oral	13
		Aripiprazole INJ	2
		Aripiprazole oral	12
		Chlorpromazine oral	0
		Flupenthixol INJ	1
		Haloperidol INJ	0
		Haloperidol oral	1
		Olanzapine oral	4
		Paliperidone INJ	5
		Paliperidone oral	1
		Quetiapine oral	0
		Risperidone INJ	1
		Risperidone oral	6
		Sulpride oral	0
		Trifluoperazine oral	1
		Zuclopenthixol INJ	4

Abbreviations: INJ, Injection; LAI, Long acting Injection.

## 4 DISCUSSION

The main finding of this paper is that the prevalence of clozapine use is just 0.038%. Bachmann et al. propose a prevalence of 0.2% (200/100,000) as the ideal goal for percentage of individuals on clozapine in the population. This aspirational target is based on prevalence figures of 0.5%–0.7% for worldwide prevalence of schizophrenia and one third of these suffering from TRS. The prevalence of clozapine utilization in Qatar, therefore, is one of the lowest in the world within the published literature, around 20‐fold less than aspirational target.

Clozapine was licensed for use in Qatar and has been available in the national formulary since 2006. Although plasma clozapine monitoring is not yet available routinely for patients on clozapine in Qatar, the MTM clinic follows robust clinical guidelines that are based upon NICE and Maudsley guidelines for clinical monitoring of patients. These guidelines are not as prohibitively restrictive as those in Japan, which is the only country with comparably low clozapine treatment prevalence (Bachmann et al., 2017). In fact, countries with similar guidelines appear to have much higher prescription rates compared to Qatar (Bachmann et al., 2017). Additionally, the data in this study show that clozapine prescription in Qatar follows the guidelines rather faithfully and over 90% of the patients have an established diagnosis of schizophrenia and more than 80% of patients have had trials of two or more antipsychotics. So, the absence of plasma‐level monitoring is unlikely to explain this huge deficit in clozapine prescribing fully.

A possible explanation arises when the differences in the nationality of individuals on clozapine in Qatar are considered. Being a migrant majority country, native Qataris constitute only 10% of the population, yet comprise just over half (53%) of all the individuals on clozapine. The percentage of native Qataris on clozapine when calculated against their own population size (300,000) within the whole population of Qatar yields a prescription rate of 0.18%, which is not very far off from the aspirational goal.

The migrant population in Qatar are derived from across the world but a significant majority constitute manual and craft workers from the Indian subcontinent (Snoj, 2019). Prevalence studies of common mental health disorders within these groups are lacking and a major reason for which is the transient nature of this population. It is assumed that prevalence of TRS is likely to be lower in this immigrant group than general population levels as functioning economic immigrants are a self‐selecting group without major chronic debilitating and relapsing illnesses.

Additionally, should individuals within these groups present with TRS, it is likely that they will return to their home countries as they cannot sustain economic activity and do not stay in Qatar for extended periods that would allow for adequate antipsychotic trials and monitoring that is needed for clozapine initiation. The fact that there is near absence of any individuals from the Indian subcontinent in the cohort gives more credence to this assumption. Also, clinician hesitation over initiating clozapine when they are not aware of the monitoring facilities in the individual's home country could be another issue that restricts prescriptions.

Our study population was diagnosed with TRS and appropriately prescribed clozapine; however, only 12% had tried two antipsychotic medications, in concordance with international guidelines, prior to initiation of clozapine and the majority (68%) had tried three or more agents. The antipsychotic groups trialed prior to clozapine included oral FGAs (57%), oral SGAs (81%), and depot long acting injection (LAI) (68%). These findings are suggestive of the physicians’ hesitancy to start patients on clozapine and continue to treat it as a last‐resort treatment option. Multiple studies have indicated delay in the initiation of clozapine after diagnosis of TRS (Mortimer et al., 2010; Taylor et al., 2003; Wheeler, 2008).

From those who had been initiated on clozapine, it was observed that 81% had been taking clozapine for 5 years or longer, and about 88% had been taking clozapine for 2 years or longer. Previous data from the European countries revealed similar trends that show that maintenance rates for clozapine are higher than those for SGAs (i.e., risperidone, olanzapine, quetiapine) (Ciudad et al., 2008; Taylor et al., 2008). This may possibly be due to the frequent contact of the patient with the healthcare workers in order to perform the needed blood monitoring while on clozapine therapy. Our data demonstrated a relatively high rate of clozapine monotherapy (58%) than that shown in the literature, though one study demonstrated clozapine monotherapy rates as high as 89% (Wheeler, 2008). Positive therapy outcomes for patients on clozapine could not be measured as we extracted the collected data retrospectively. Nonetheless, we can assume that the patients were well maintained on clozapine monotherapy. However, clozapine monotherapy was not sufficient for some patients and hence augmentation with other antipsychotics was needed for maintenance. The patients who were on augmentation therapy were lower (42%) in our study population than reports in the literature where up to 50% of patients affected by TRS were not managed by clozapine alone and required combination therapy (Porcelli et al., 2012). From the concomitant antipsychotics, Amisulpride and Aripiprazole (12% and 13%) were mostly used for augmentation and in 13% of the patients, the augmenting agent was long‐acting injectable antipsychotic.

Another finding of the study was that the average dose of clozapine was 325 mg/day across the cohort. There have been debates over whether individuals of Asian ethnicity require lower doses of clozapine when compared to Caucasians (de Leon et al., 2020). The individuals in this study were, on average, receiving higher doses of clozapine than in studies from other Asian countries. Although potentially interesting, in the absence of history of smoking and actual plasma clozapine levels, the significance of this finding cannot be discussed fully at this time.

Our results are limited by the accuracy and completeness of the information contained in the medical charts especially for patients who started clozapine before the implementation of the electronic medical record Cerner or who initiated clozapine abroad. It is hoped that this study will enable implementation of clozapine plasma‐level monitoring in Qatar and help in capturing more comprehensive data about patients on clozapine. Another limitation to study is the small sample size; however, the observational approach used provides real‐world information regarding the use of clozapine in a Qatar population.

Overall, this is a significant first study of its kind in the MENA region that starts to explore this important topic within the unique sociocultural milieu of Qatar. It highlights areas of good practice and sets foundation for more directed studies in this field that can be carried out in an informed manner.

## CONFLICT OF INTEREST

The authors declare no conflict of interest.

### PEER REVIEW

The peer review history for this article is available at https://publons.com/publon/10.1002/brb3.2617.

## Data Availability

Any additional data are available from the corresponding author on a reasonable request.
